# Self-harm in Young People: Investigating the Role of Resilience and Posttraumatic Stress Related to the COVID-19 Pandemic

**DOI:** 10.1007/s40653-022-00511-z

**Published:** 2022-12-28

**Authors:** Finiki Nearchou

**Affiliations:** School of Psychology, University College Dublin, Newman Building, Belfield Campus, Dublin, Dublin 4 Ireland

**Keywords:** Resilience, Pandemic, Coronavirus, Self-harm, Non-suicidal self-injury, Depression, Posttraumatic

## Abstract

Evidence shows that young people may have experienced increased levels of posttraumatic stress and depression during the COVID-19 pandemic. However, the landscape on self-harm is still unclear. This study aimed to examine the role of COVID-19 related posttraumatic stress, depression and resilience as predictors of self-harm with and without suicidal intent. Participants were 625 young people aged 17–25 years old (*M* = 20.2 years, *SD* = 2.47). Resilience was measured using the self-reported Child & Youth Resilience Scale Measure – Revised (CYRM-R). Posttraumatic stress related to COVID-19 were measured using the Impact of Event Scale- Revised. Depression was measured using the depression subscale of the Depression, Anxiety and Stress Scale–21. Self-harm was evaluated with two dichotomous items. Participants reported high levels of depression and COVID-19 posttraumatic stress, and a significant percentage reported engaging in self-harm. Hierarchical logistic regressions showed that caregiver resilience decreased approximately 20% the odds of engaging in self harm with and without suicidal intent remaining a consistent predictor even after accounting posttraumatic stress and depression in the models. Posttraumatic stress and depression predicted a one-fold increase in the odds of engaging in self-harm with and without suicidal intent. However, posttraumatic stress was no longer a significant predictor when depression was entered in the model in self-harm without suicidal intent. The COVID-19 pandemic may have increased the likelihood of engaging in self-harm in young people. However, caregiver resilience seems to operate as a protective factor. This important finding carries implications beyond the context of the COVID-19 pandemic.

## Introduction

### The COVID-19 Pandemic

COVID-19 is an infectious respiratory disease caused by the SARS-CoV-2 virus, which has resulted in over 574 million confirmed cases of COVID-19 and in over six million deaths as of 31^st^ of July 2022 (World Health Organization, 2022). Numerous public health restrictions such as limits on gatherings, restriction of movements, and closures of schools and businesses were imposed globally by governments to contain the spread of the virus. Although the health risks of infection were greater for older adults, the impact of distress and concern of infection of self and loved ones, the impact of lockdowns and quarantine, and the disruption of daily life was a shared adversity across all age groups. Certain adversities, such as the disruption to school, university, and loss of employment have disproportionately impacted young people (International Labour Organization, 2020). Young people may have also been impacted through traumatic events such as the death of a parent or a caregiver from COVID-19 (Ahmann, 2021) with estimates showing that as many as 1.5 million young people worldwide between March 2020 and April 2021 may have experienced the death of a primary or secondary caregiver from COVID-19 (Hillis et al., 2021).

### The Impact of COVID-19 on Young People

Adolescence and young adulthood are characterised by transitions and have been associated with many challenges in relation to mental health and well-being. Many mental health disorders diagnosed later in adulthood have their onset during adolescence and/or young adulthood. Thus, it is not surprising that early in the COVID-19 pandemic scholars have expressed concerns about its potential impact on youth mental health (Ghosh et al., 2020; Wang et al., 2020).

Indeed as of to date there is evidence indicating the mental health impact of the pandemic on youth (Nearchou et al., 2020), with research specifically highlighting this impact on cohorts in late adolescence (i.e. emerging adults) usually aged between 18–25 years old. The impact of COVID-19 on the mental health of emerging adults has been well documented by multiple studies and reviews (e.g., Panchal et al., 2021; Racine et al., 2021; Zhen & Zhou, 2022). For instance, cross-sectional international evidence showed that emerging adults were more likely to experience higher levels of stress and anxiety than older adults (Varma et al., 2021). Recent meta-analytic evidence indicated that emerging adults exhibited higher prevalence rates of anxiety/depressive symptoms, sleep problems, and suicidal ideation (Dragioti et al., 2022).

Self-harm can be described as an act of deliberately initiating a behaviour (e.g., self-cutting) driven by an individual’s intention to cause harm to themselves (Madge et al., 2008); another term commonly used in the literature for the same phenomenon is non-suicidal self-injury. Self-harm without suicidal intent is usually distinguished from self-harm with at least some suicidal intent and the latter may as well be classified as suicidal behaviour (Nock et al., 2008). Self-harm presents at high rates among young people and has been suggested as a strong risk factor predicting death by suicide, with suicide being the leading cause of death in this age-group (Patel et al., 2007). Self-harm is ranked as one of the top 10 causes of years lost to disability for young people aged 10 to 24 years (Gore et al., 2011) and carries high risks of suicide (Beckman et al., 2016). Self-harm with and without suicidal intent in young people has been identified as a longitudinal predictor of detrimental psychosocial outcomes (Mars et al., 2014).

Exposure to traumatic events and posttraumatic stress disorder (PTSD) symptoms have been associated with self-harm in emerging adults (Ennis et al., 2020). It has been suggested that self-harm may operate as a maladaptive coping strategy in the light of trauma exposure, while trauma symptoms mediate the relationship between trauma exposure and self-harm (Smith et al., 2014). Given the duration, severity and intensity of the global public health crisis induced by the COVID-19 pandemic, it is not surprising that there is some research reporting an increase in self-harm in young people during the course of the pandemic (Du et al., 2021). However, despite reports of higher rates of suicidal behaviours including self-harm during the pandemic, the low quality of studies included in relevant systematic reviews warrants caution in interpreting findings (Farooq et al., 2021). This, in conjunction with the inconclusive findings of engagement rates of young people in self-harm with and without suicidal intent during the COVID-19 pandemic (Zolopa et al., 2022), highlights the need to obtain more evidence on this matter.

### Resilience

Resilience is a complex dynamic process that leads to adaptation and change when a system (e.g., a forest or a human being) is exposed to stress. Informed by a socio-ecological framework, resilience relies on the capacity of a biopsychosocial system such as in case of human resilience, a family, a person, or a community, to navigate towards resources that promote adaptation and sustain well-being. In this framework of a socio-ecological operationalisation, resilience also relies on the capacity of the systems to create a fertile ground, which will enable individuals to access those resources in meaningful, culturally and contextually, manners (Ungar, 2011).

Individual characteristics as well as family and other caregiver supports have been well documented in the literature as protective factors that promote resilience in young people exposed to adversities including traumatic experiences (Hsieh et al., 2016; Nearchou, 2018; Ungar, 2008). A recent meta-analysis that synthesized evidence from the past two decades identified support from family as a significant protective factor in children and adolescents exposed to trauma (Xiong et al., 2022). Positive family relationships have been also suggested as to buffer young adults against the effects of trauma experience as young adults may be more likely to seek help from parents or family in general when exposed to trauma (Wang et al., 2021).

Similar to evidence in adult cohorts (e.g. Nearchou & Douglas, 2021), there is some evidence suggesting the potential buffering effect of resilience against the mental health impact of the pandemic in emerging adults (Li et al., 2021; Shanahan et al., 2022). Personal competence and support from family as factors that promote resilience, have been associated with lower levels of depression, anxiety and PTSD symptoms in emerging adults during the course of the COVID-19 pandemic (Liu et al., 2020; Noh & Park, 2022).

Although research on youth self-harm and resilience is quite limited, yet there is some evidence suggesting that emerging adults who engage in self-harm tend to report lower resilience levels than their counterparts with no such history (Watson & Tatnell, 2022). With regard to specific personal and relational protective factors, personal competence has been reported as a factor associated with resilience in emerging adults who self-harm within a context of violence (Huang & Mossige, 2015). Positive family environment such as perceived support from family and family cohesion features among protective factors identified to mitigate the risk of self-harm in young people (Khan & Ungar, 2021).

### The Present Study

The COVID-19 pandemic has had a negative impact on youth mental health, while it has been suggested that many young people may experience posttraumatic stress related to this public health crisis. In the context of the present study, posttraumatic stress is operationalised as young people’s traumatic stress responses to the COVID-19 pandemic. In order to better support young people in the aftermath of this pandemic, it is essential to identify the impact of posttraumatic stress related to COVID-19 given their susceptibility to mental health difficulties. Furthermore, because young people are at increased risk of engaging in self-harm and considering the links between posttraumatic stress, depression and self-harm, it is important to deepen our understanding on this matter. Research shows that young people exposed to traumatic events, including the COVID-19 pandemic, may present with co-occurring mental health difficulties such as depression and posttraumatic stress (Cénat & Derivois, 2015; Liu et al., 2020; Nearchou et al., 2020). As such, the present study aimed to examine posttraumatic stress levels and depression levels as two distinct, yet co-occurring predictors of self-harm in a sample of young people.

Clarifying the role of resilience in the potential pattern of associations between depression, posttraumatic stress and self-harm can be valuable in informing the design of prevention and/or intervention programmes targeting young people at risk of self-harm. Thus, resilience was included as a potential predictor of the likelihood of self-harm in young people of this sample. Finally, while self-harm with and without suicidal intent are perceived as two different types of self-harm in the literature, yet these phenomena have scarcely been examined independently from one another in the same cohort of young people. In addition, existing evidence on the association between resilience and self-harm in young people is extremely limited. Thus, the present study aimed to add to our knowledge by examining the predictive role of resilience separately in self-harm without suicidal intent and in self-harm with suicidal intent.

## Method

### Participants

Data of this research derived from a larger cross-sectional nationwide study (The Your Youth Health Project) conducted to explore the impact of COVID-19 on the mental health of young people in the Republic of Ireland (Nearchou et al., 2022). The sample of the present study comprised young people (N = 625) aged 17 to 25 years old (*M* = 20.2 years, *SD* = 2.47). Most participants identified as females (80%) and Irish (82%). Over half of participants (62%) were enrolled in third-level education institutions at the time of data collection, 22% were secondary school students, and 12% reported being employed.

## Measures

### Resilience

Resilience was measured using the self-reported Child & Youth Resilience Scale Measure – Revised (CYRM-R) (Resilience Research Centre, 2018). The CYRM-R was developed to measure the construct of resilience through a socio-ecological framework. This widely used tool has demonstrated excellent psychometric properties and has been recommended for use across diverse cultures and contexts (Jefferies et al., 2019). The CYRM-R includes 17 items asking participants to rate the extent to which each statement measuring resilience applies to them. The tool comprises two subscales reflecting caregiver resilience (seven items) and personal resilience (10 items). Example items are ‘I get along with people around me’ (personal resilience) and ‘I talk to my family/caregiver(s) about how I feel (for example when I am sick or have done something wrong)’ (caregiver resilience). For the present study we used the three-point simplified version of the tool, with each item scored on a Likert scale ranging from 1 (‘No’) to 3 (‘Yes’). Each subscale yields a sum score of resilience ranging from 10–30 for personal resilience and from 7–21 for caregiver resilience, with higher scores indicating higher levels of the respective resilience construct. The reliability coefficient both for caregiver (*α* = 0.84) and personal resilience (*α* = 0.77) subscales was excellent for the present sample.

### Post-traumatic Stress related to COVID-19

Posttraumatic stress levels related to COVID-19 were measured using the Impact of Event Scale-Revised (IES-R) (Weiss & C.R., 1997). The IES-R is one of the most widely used self-report instruments that measures posttraumatic stress levels related to experiencing a specific traumatic event in the general non-clinical population. The IES-R has been widely employed to measure posttraumatic stress levels related to the COVID-19 pandemic in non-clinical samples (e.g., Cheikh Ismail, et al., 2021; Paulino et al., 2021). The IES-R has been also used to assess posttraumatic stress related to COVID-19 in cohorts of young people (e.g. Essadek & Rabeyron, 2020; Ke et al., 2022). Recent evidence confirmed the tool’s psychometric ability to measure posttraumatic stress related to COVID-19 in large epidemiological cohorts (Aljaberi et al., 2022; Braule Pinto et al., 2022). The IES-R includes 22 items loading on three subscales and reflecting hyperarousal (seven items), intrusion (seven items) and avoidance (eight items). Although not a diagnostic tool, the IES-R was developed and validated using a specific traumatic event as a reference in the introduction to the individual within a specific time frame of the past seven days. For the purposes of the present study, we indicated the COVID-19 pandemic as the traumatic event in reference. Items are rated on a five-point Likert scale ranging from 0 (‘Not at all’) to 4 (‘Extremely’). An example item is ‘I tried not to talk about it’. The scale produces a total sum score ranging from 0 to 88, with higher scores indicating higher levels of posttraumatic stress related to COVID-19. The reliability coefficient of the tool was excellent for the present sample (*α* = 0.94).

### Depression

Depression levels were measured using the depression subscale of the Depression, Anxiety and Stress Scale–21 (DASS-21) (Lovibond & Lovibond, 1995). The DASS-21 is a well-established psychometric tool developed to measure emotional states related to depression, anxiety and stress. Evidence indicates that the tool possesses sound psychometric properties for use in non-clinical samples from the general population including adolescents and young adults (Henry & Crawford, 2011; Tully et al., 2009). The depression subscale of the DASS-21 includes seven items scored on a four-point Likert scale ranging from 0 (‘Never’) to 3 (‘Almost always’) and asks participants to rate how much each item applied to them during the past week. An example item is ‘I was unable to become enthusiastic about anything’. The total score of the depression subscale is calculated by adding the seven items and then multiplying their sum by two. The total score ranges from 0 to 42, with higher scores indicating higher levels of depression. The reliability coefficient of the depression subscale was excellent for the present sample (*α* = 0.90).

### Self-harm

Participants were presented with a set of items including two questions about whether they engaged in deliberate self-harm with and without suicidal intent: ‘Have you ever deliberately hurt yourself without wanting to take your life?’ and ‘Have you ever deliberately hurt yourself wanting to take your own life?’. The response to these items was dichotomous (yes/no). This set of items has been previously used in the same population cohort (Mahon et al., 2022).

### Procedure

Research presented in this paper employed a Patient and Public Involvement in Research approach where young people actively contributed to the research design. The study questionnaire was finalised upon consultation with the project’s youth advisory panel (Cross Care Youth Services of Dún Laoghaire). Data were collected as part of a larger project through an anonymous online survey using the Qualtrics platform between October 2020 and May 2021 mainly by using purposive sampling. Young people aged 12–25 years old residing in the Republic of Ireland were eligible to participate and were invited through different channels and networks including social media platforms. The estimated time completion of the survey was approximately 20–30 min. Informed consent was obtained from all adult participants aged 18–25 years old. Secondary school students (12–17 years old) were recruited online through schools, with the principal or guidance counsellor acting as gatekeepers and sharing the study information with parents. Secondary schools were randomly selected to represent all country areas from the list obtained by the Department of Education in the Republic of Ireland. The young people were not offered any incentives to participate in the study. For participants aged 12–17 years old, consent from a parent or guardian was initially obtained, which was followed by informed assent from the young individual. The present paper presents findings based on data collected from young people aged 17–25 years old. Ethical approval for the study was obtained by the Research Ethics Committee of the institution affiliated with the implementation of this study.

### Data Analysis Overview

Descriptive information is presented for all study variables including cut-off scores for depression and posttraumatic stress following guidelines related to the respective tools (i.e., DASS-21 and IES-R). Two hierarchical logistic regressions were applied with the outcome variable being self-harm with and without suicidal intent (coded as 1 for engaging in self-harm and 0 for not engaging in self-harm). Personal and caregiver resilience were entered as predictors in step 1, COVID-19 related posttraumatic stress was entered as predictor in step 2 and depression was entered as predictor in step 3 of the hierarchical regression models. This allowed to examine the role of resilience while considering additional contributing variables in predicting the likelihood to engage in self-harm with and without suicidal intent. Alpha was set at 0.05 with a 95% confidence interval. Likelihood Ratio chi-square test (*χ*^*2*^) was used to estimate model improvement in each step of adding predictors with a significant p-value (< 0.05) indicating a significant improvement. The Nagelkerke Pseudo *R*^*2*^ index ranges from 0 to 1 and was used to estimate the null deviance accounted for by the predictors. Analyses were performed using IBM SPSS software version 27.

## Results

### Descriptive Statistics and Intercorrelations

Table 1 presents frequencies of self-reported self-harm, and levels of depression and COVID-19 posttraumatic stress in young people. A little less than half of this sample (45%) reported engaging in self-harm without suicidal intent and a little under 20% reported engaging in self-harm with suicidal intent. Levels of depression and COVID-19 posttraumatic stress levels were calculated using the cut-off scores for DASS-21 and IES-R respectively (see Table 1). Approximately 20% of the sample reported normal levels of depression, while the majority reported experiencing either mild, moderate, severe or extremely severe levels. Notably, 27% reported depression levels that were classified under the highest end of the continuum. Young people reported high levels of COVID-19 posttraumatic stress with only a little over one third of the sample being classified under the normal range of the continuum. Notably, 36% reported levels of posttraumatic stress falling under the highest end of the continuum.

**Table 1:** Levels of depression and posttraumatic stress symptoms related to COVID-19, and frequencies of self-harm in the study sample

	Normal (n) %	Mild (n) %	Moderate (n) %	Severe (n) %	Extremely Severe (n) %
Depression	(124) 19.8%	(77) 12.3%	(157) 25.1%	(101) 16.2%	(166) 26.6%
COVID-19 PTS	(233) 37.3%	(103) 16.5%	(67) 10.7%	(222) 35.5%	
	**No** n (%)	**Yes** n (%)			
Self-harm without suicidal intent	(347) 55.5%	(278) 44.5			
Self-harm with suicidal intent	(514) 82.2%	(111) 17.8%			

COVID-19 related posttraumatic stress (COVID-19 PTS) measured by the Impact of Event Scale-Revised and depression measured by the respective subscale of the Depression Anxiety and Stress Scale-21*. Depression cut-off scores:* normal (0–9), mild (10–13), moderate (14–20), severe (21–27), extremely severe (≥ 28)*. PTS cut-off scores:* normal (0–23), mild (24–32), moderate (33–38), severe (≥ 39)

Table 2 presents correlations among personal and caregiver resilience, depression and COVID-19 posttraumatic stress. All variables were significantly correlated. Caregiver resilience showed a positive moderate correlation with personal resilience, a negative moderate correlation with depression and a negative weak correlation with COVID-19 posttraumatic stress. Personal resilience showed negative correlations with depression (moderate) and COVID-19 posttraumatic stress (weak), while depression and COVID-19 posttraumatic stress were moderately and positively correlated.

**Table 2:** Mean scores, standard deviations ( ±) and bivariate correlations for resilience, depression and COVID-19 related posttraumatic stress (PTS)

Variable	1	2	3	Mean	±
1. Caregiver Resilience				17.9	3.007
2. Personal Resilience	0.53*	-		16.3	3.18
3. Depression	-0.37*	-0.44*	-	19.6	11.1
4.COVID-19 PTS	-0.25*	-0.30*	-0.58*	31.8	19.2

**p-value < 0.001

### Self-harm without Suicidal Intent

Findings of the logistic regression for engaging in self-harm without suicidal intent are presented in Table 3. Upon adding personal and caregiver resilience (step 1) the model was significantly improved (Likelihood Ratio) *χ*^*2*^ (2) = 98.1, *p* < 0.001 with 19.4% of the null deviance accounted for by the set of predictors. In this step both personal (OR = 0.93) and caregiver resilience (OR = 0.79) decreased the likelihood of a young individual engaging in self-harm without suicidal intent. The model further improved *χ*^*2*^ (3) = 113.3, *p* < 0.001 upon adding COVID-19 posttraumatic stress as a predictor with 22.2% of the null deviance accounted for by the set of three predictors. Young people who experienced higher levels of posttraumatic stress were a little over one time more likely to engage in self-harm without suicidal intent (OR = 1.02). However, only caregiver resilience remained a significant predictor of self-harm. Finally, when depression was added (step 3) as a predictor, the full model was further improved *χ*^*2*^ (4) = 132.2, *p* < 0.001. The Nagelkerke *R*^*2*^ index indicated that 26% of the null deviance accounted for by the set of four predictors. Depression predicted an increased likelihood of engaging in self-harm above and beyond resilience and COVID-19 posttraumatic stress (OR = 1.05). Upon adding depression to the model only caregiver resilience remained a significant predictor, while personal resilience and posttraumatic stress no longer significantly contributed to the model.

**Table 3:** Hierarchical logistic regression for predictors of likelihood to engage in self-harm without suicidal intent

Step	Predictor	*B*	SE*B*	Exp*(B)*	*p*- value	CI 95% Lower	CI 95% Upper
1							
	Personal Resilience	-0.08	0.04	0.93	0.02	0.87	0.98
	Caregiver Resilience	-0.24	0.04	0.79	< 0.001	0.73	0.85
2							
	Personal Resilience	-0.05	0.03	0.95	0.13	0.89	1.02
	Caregiver Resilience	-0.23	0.04	0.79	< 0.001	0.74	0.85
	COVID-19 PTS	0.02	0.005	1.02	< 0.001	1.009	1.03
3	Personal Resilience	-0.02	0.04	0.98	0.65	0.92	1.06
	Caregiver Resilience	-0.22	0.04	0.80	< 0.001	0.75	0.88
	COVID-19 PTS	0.006	0.006	1.006	0.28	0.99	1.02
	Depression	0.05	0.01	1.05	< 0.001	1.02	1.07

*SE* standard error, *CI* confidence interval, *COVID-19 PTS* COVID-19 related posttraumatic stress

### Self-harm with Suicidal Intent

Findings of the logistic regression for engaging in self-harm with suicidal intent are presented in Table 4. Upon adding personal and caregiver resilience (step 1) the model was significantly improved (likelihood ratio) *χ*^*2*^ (2) = 87.9, *p* < 0.001 with 21.6% of the null deviance accounted for by the set of two predictors. In this step both personal (OR = 0.89) and caregiver resilience (OR = 0.78) decreased the likelihood of a young individual engaging in self-harm with suicidal intent. The model further improved *χ*^*2*^ (3) = 117.7, *p* < 0.001 upon adding COVID-19 posttraumatic stress as a predictor with 28.3% of the null deviance accounted for by the set of three predictors. Young people who experienced higher levels of COVID-19 posttraumatic stress were a little over one time more likely to engage in self-harm with suicidal intent (OR = 1.03). However, only caregiver resilience remained a significant predictor of self-harm. Finally, when depression was added (step 3) as a predictor, the full model was further improved *χ*^*2*^ (4) = 127.8, *p* < 0.001. The Nagelkerke *R*^*2*^ index indicated that 30% of the null deviance accounted for by the set of four predictors. Depression predicted an increased likelihood of engaging in self-harm above and beyond resilience and COVID-19 posttraumatic stress (OR = 1.04). Upon adding depression to the model, caregiver resilience (OR = 0.81) and posttraumatic stress (OR = 1.02) remained significant predictors of engaging in self-harm with suicidal intent.

**Table 4:** Hierarchical logistic regression for predictors of likelihood to engage in self-harm with suicidal intent

Step	Predictor	*B*	SE*B*	Exp*(B)*	*p*- value	CI 95% Lower	CI 95% Upper
1							
	Personal Resilience	-0.11	0.04	0.89	0.003	0.83	0.96
	Caregiver Resilience	-0.24	0.04	0.78	< 0.001	0.73	0.85
2							
	Personal Resilience	-0.08	0.04	0.93	0.06	0.86	1.002
	Caregiver Resilience	-0.23	0.04	0.80	< 0.001	0.74	0.86
	COVID-19 PTSS	0.04	0.006	1.03	< 0.001	1.02	1.03
3	Personal Resilience	-0.04	0.04	0.95	0.28	0.88	1.04
	Caregiver Resilience	-0.21	0.04	0.81	< 0.001	0.75	0.88
	COVID-19 PTSS	0.02	0.01	1.02	< 0.001	1.009	1.04
	Depression	0.04	0.02	1.04	0.002	1.02	1.07

*SE* standard error, *CI* confidence interval

## Discussion

This is the first study to examine the role of resilience, posttraumatic stress levels related to COVID-19 and depression levels as predictors of self-harm with and without suicidal intent in young people aged 17 to 25 years old. A little under 50% of our sample reported engaging in self-harm without suicidal intent, while 18% reported engaging in self-harm with suicidal intent. Both caregiver and personal resilience were significant predictors of decreased likelihood to engage in self-harm with and without suicidal intent without considering any other predictors. Interestingly, caregiver but not personal resilience remained a significant predictor in both regression models when added COVID-19 posttraumatic stress in the models (step 3), suggesting that caregiver resilience did play a significant role in predicting self-harm during the pandemic in young people. Depression predicted an increased likelihood to engage in self-harm with and without suicidal intent above and beyond resilience and COVID-19 related posttraumatic stress levels. When depression was included as a predictor, posttraumatic stress levels did no longer contributed to predicting self-harm without suicidal intent. This indicates that depression remains the most significant predictor of self-harm in young people despite experiencing posttraumatic stress levels related to the COVID-19 pandemic.

A significant percentage of young people in our sample reported engaging in self-harm with (18%) and without suicidal intent (45%). This finding is overall consistent with existing international evidence on self-harm rates in young people (Brunner et al., 2014). Approximately 18% of the present sample reported engaging in self-harm with suicidal intent, which is similar with findings reported by other studies conducted during the pandemic (Ellis et al., 2020). However, rates in the present study are slightly elevated when compared with a pre-pandemic study in 8,290 young adults from Ireland which reported that 38% and 12% of the sample engaged in self-harm without and with suicidal intent respectively (Dooley et al., 2019). Given the impact of the pandemic on youth mental health, it is not surprising that levels of self-harm appear somewhat elevated when compared to pre-pandemic levels. While young people of the present sample may have engaged in self-harm before the COVID-19 outbreak, detected levels in this study may appear elevated because mental health services were significantly interrupted by prolonged lockdowns, which have been a barrier for seeking help (Sass et al., 2022). Thus, young people who could not access their standard level of care and support may engaged more frequently in self-harm. Ultimately, this finding suggests that self-harm rates in this cohort remain high and may be even more elevated over the course of the pandemic. It also indicates that self-harm regardless of suicidal intent remains a serious youth mental health problem placing young people at increased risk of suicidal behaviour (Kaess, 2022).

Overall, the hierarchical logistic regression models highlighted that resilience decreases the risk of self-harm regardless of suicidal intent in this age group (17–25 years old) of young people. This corroborates existing findings regarding the association between resilience and self-harm in youth (e.g. Lai et al., 2021). However, different aspects of resilience i.e., personal and caregiver resilience yielded different patterns of associations with self-harm. Caregiver resilience was the most consistent predictor of the decreased likelihood of self-harm (i.e., 20% decrease in the odds of engaging in self-harm) regardless suicidal intent even after entering COVID-19 related posttraumatic stress levels and depression in the models. This is in keeping with evidence indicating positive family context as a protective factor against self-harm in young people (Gámez-Guadix et al., 2022; Khan & Ungar, 2021; Tian et al., 2019). Family (caregiver) support has been recognized as an important protective factor associated with better mental health outcomes in youth exposed to adversities (Theron et al., 2022). Conversely, personal resilience demonstrated a mixed predictive role in self-harm within and across the two hierarchical regression models. While personal resilience was an initial significant predictor of self-harm regardless suicidal intent, it was no longer significant when COVID-19 related posttraumatic stress levels and depression were considered as predictors. Although individual qualities may be associated with resilience in youth, in the context of a pandemic family resilience seems to supersede any individual-related contribution to decreasing self-harm. The COVID-19 pandemic encompassed prolonged lockdowns and disruption of daily activities, while restrictions in social life brought loneliness. Thus, it is not surprising that engagement with family was deemed important for young adults (Blackwell et al., 2022; Theron et al., 2021), and especially for those with mental health needs that emerged during the pandemic (Marchini et al., 2021).

The present study showed that COVID-19 related posttraumatic stress levels increased one-fold the risk of engaging in self-harm with and without suicidal intent in young adults. This finding is consistent with existing evidence indicating that posttraumatic stress is a predictor of self-harm (Dixon-Gordon et al., 2014; Ennis et al., 2020). However, posttraumatic stress levels specifically related to the pandemic was not a consistent predictor across the two different types of self-harm (with and without suicidal intent) in emerging adults. Interestingly, depression increased the odds in engaging with self-harm without suicidal intent above and beyond the contribution of posttraumatic stress. This suggests that for emerging adults of this sample who experienced depression and COVID-19 related posttraumatic stress levels, the former may have played a significant role in increasing the odds for self-harm. Depression has been one of the most well-established markers of self-harm in young people (Tuisku et al., 2014; Zubrick et al., 2017), which despite posttraumatic stress levels induced by a global health crisis, may still remain the most significant predictor of self-harm. However, because the temporal measurement of depression in the present study was confined to a specific timeframe, it is impossible to know whether depression was a pre-existing condition for some young adults of this sample.

A different pattern of findings emerged for self-harm with suicidal intent showing that both COVID-19 related posttraumatic stress levels and depression increased the odds of self-harm for young people who wanted to take their life. This finding is consistent with evidence showing that posttraumatic stress and depression are identified risk factors of suicidal behaviour (Panagioti et al., 2012). Interestingly, this finding also highlights that for some young people the impact of the pandemic may have been traumatic and may have exacerbated existing depression symptoms, thus increasing the likelihood to engage in self-harm with intention to take their life.

As Table 1 shows, approximately two out of three emerging adults of the present study (63%) reported levels of COVID-19 related posttraumatic stress falling under the cut-off classification that indicates a clinical PTSD concern (i.e., > 22 score). Approximately one out of three (35.5%) young adults reported levels classified under the severe end of the continuum. Taken together, these results indicate that young people may have experienced elevated posttraumatic stress levels specifically linked to the pandemic. This corroborates existing evidence in this age cohort (Chi et al., 2020; Liu et al., 2020; Ochnik et al., 2021). Young adults reported high levels of depression with 27% classified under the highest end of the continuum. Although consistent with international pandemic-related evidence (Chi et al., 2020; Liu et al., 2020; Ochnik et al., 2021), these levels are considerably higher when compared to pre-pandemic levels in young adults from Ireland (see Dooley et al., 2019).

Data for this research were collected over a period of prolonged and strict lockdowns (October 2020-May 2021). Since March 2020, the Republic of Ireland has experienced three lockdowns, with the third (January–May 2021) placing the country very high on the list of the COVID-19 Containment and Health Index. This index is calculated on the basis of response metrics such as school closures, travel bans, restrictions of internal movements, and workplace closures (Hale et al., 2021). These prolonged lockdowns may have impacted different aspects of young people’s lives. For instance, young people in Ireland have disproportionally suffered from job losses (Roantree et al., 2021), while economic and social changes have impacted romantic and sexual relationships as well as access to confidential and affordable health care services (Lindberg et al., 2020). Most young people abandoned plans of travelling after college and some reported loss of motivation and confidence to start job searching (Timonen et al., 2021).

### Implications

Findings of this study have important implications for research and practice. A significant proportion of emerging adults of this sample reported engaging in self-harm. However, the true rates of self-harm maybe even higher. This realisation becomes increasingly important especially considering that during the first months of the pandemic the strict lockdown measures have disrupted mental health services and supports, which in turn imposed barriers for those who self-harm to access help (Sass et al., 2022). These lockdown measures may have masked the true mental health burden in young people during the pandemic (Kaess, 2022). Self-harm has been suggested as a marker of suicide risk and general mental health in youth, while there are concerns that the mental health impact of the pandemic is still unravelling.

Evidence from past epidemics indicated that it was in the years following the epidemics that there was a noticeable increase in suicidal behaviour (Zortea et al., 2021).Thus, in the aftermath of the COVID-19 pandemic, we need to continue expanding our knowledge on self-harm as a potential increase in self-harm can be linked to an increase in suicide risk. We also need to obtain more evidence-based knowledge on the role of resilience in mitigating the potential risks of self-harm associated with this pandemic in young people.

Findings of the present study significantly add to the notably limited pool of evidence regarding the role of resilience in youth self-harm carrying implications beyond the context of the COVID-19 pandemic. Resilience is associated with individual qualities as well as with resources located in systems (ecologies) surrounding the individual, such as family, school, community and health services. In this study, caregiver resilience consistently predicted a 20% decrease in the odds of engaging in self-harm with and without suicidal intent in young adults regardless of COVID-19 posttraumatic stress and depression. Viewed though a socio-ecological lens, resilience is inextricably linked to the systems surrounding the individual. Subsequently, this important finding indicates that empowering caregivers/families through targeted programmes may indirectly foster resilience through empowering emerging adults at risk, which in turn may operate as a buffer against self-harm. For instance, addressing information needs of parents (e.g., where/how to access help) whose child has engaged in self-harm, may enable parents to better support their child and promote resilience.

### Limitations

Despite its strengths the present study has some limitations that should be considered when interpreting results. First, due to a small proportion of the sample identifying as male, the present study did not explore gender differences, which warrants caution in making cross-gender inferences. However, because there is evidence suggesting that males may differ from females in self-harm behaviours regrading frequency, severity, causality and means to inflict harm, gender differences should be examined by future research (Fitzgerald & Curtis, 2017).

Second, despite comparing findings of the present study with findings in same age group individuals sampled from within the same cultural context (Dooley et al.,. 2019), the cross-sectional study design does not allow to longitudinally compare self-harm and mood changes thus capturing both the pre-pandemic and the post-pandemic era. However, because these findings significantly extend our knowledge on the role of resilience and self-harm, and because the impact of the pandemic is still unravelling, future research should further explore this field ideally by employing longitudinal designs. Third, emerging adults of this study were asked by two single items to indicate whether they have engaged in self-harm with and without suicidal intent. While information obtained allowed to identify the presence or absence of self-harm as well as to conduct analyses for this research, using a validated psychometric tool with multiple items could offer a more holistic picture of self-harm behaviour. Finally, posttraumatic stress in the present study was measured using the self-report IES-R, which is a psychometric instrument widely used as a measure of posttraumatic stress levels in non-clinical samples. While employing this tool offered an insight of the traumatic stress levels experienced by young people of this study during the COVID-19 pandemic, results should be interpreted with caution as the IES-R is not a diagnostic tool.

## Conclusions

The present study showed that a significant proportion of young people reported high levels of COVID-19 related posttraumatic stress levels and depression over the course of the pandemic. COVID-19 related posttraumatic stress levels increased the likelihood of engaging in self-harm, which highlights that the pandemic had a traumatic imprint for some young people. Caregiver resilience remained a significant predictor of a decreased likelihood in engaging in self-harm even when accounting for posttraumatic stress and depression. This indicates that resilience in young people who may be at risk for self-harm can be promoted through empowering caregivers and families to support their children in need. It also indicates that implications of this research may go beyond the COVID-19 pandemic and can be used to inform the design of future research and practice in the field of youth resilience and self-harm.

## Data Availability

Data are not available for this research.
